# Integrating AlphaFold pLDDT Scores into CABS-flex for enhanced protein flexibility simulations

**DOI:** 10.1016/j.csbj.2024.11.047

**Published:** 2024-11-30

**Authors:** Karol Wróblewski, Sebastian Kmiecik

**Affiliations:** Biological and Chemical Research Center, Faculty of Chemistry, University of Warsaw, Zwirki i Wigury 101, Warsaw 02–089, Poland

**Keywords:** Structure-based model, Protein structure, Protein flexibility, Molecular dynamics

## Abstract

CABS-flex is a well-established method for fast protein flexibility simulations, offering an effective balance between computational efficiency and accuracy in modeling protein dynamics. To further enhance its predictive capabilities, we propose incorporating AlphaFold's predicted Local Distance Difference Test (pLDDT) scores into CABS-flex simulations. The pLDDT scores, which reflect the confidence of AlphaFold's structural predictions, were integrated with secondary structure information to refine the restraint schemes used in the simulations. We tested this approach on the ATLAS database, which includes molecular dynamics (MD) simulations of nearly 1400 proteins. The results showed improved alignment of flexibility predictions with the MD data compared to previous restraint schemes. The integration of pLDDT scores also offers a new perspective on protein flexibility by incorporating structural confidence into the analysis. This development enhances the utility of CABS-flex for investigating protein dynamics and motion.

## Graphical Abstract

**Figure:**
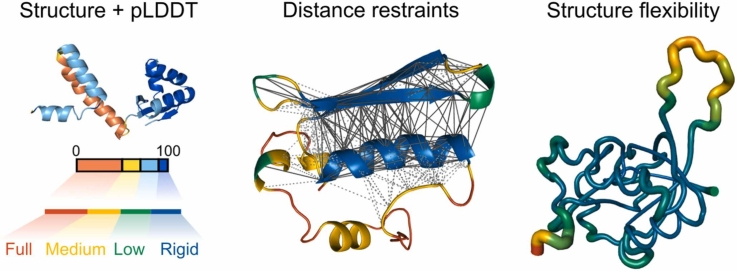

## 1 Introduction

The recent advancements driven by AlphaFold2 have revolutionized the field of structural biology, allowing researchers to predict protein structures with unprecedented accuracy [1]. While these models have largely solved many challenges in static protein structure prediction, the limitation of rigid, static snapshots is increasingly evident [2]. Understanding the dynamic nature of proteins, including their conformational flexibility and alternative biologically relevant states, is crucial for advancing both fundamental research and therapeutic applications. Therefore, there is a growing need to explore conformational ensembles that capture the full range of protein motion and biological functions. Careful integration of AlphaFold-generated models with experimental data and simulation tools is essential for developing accurate hypotheses, as the predicted models should not be viewed as absolute representations of protein structures, but rather as one of many potential configurations within a dynamic ensemble [2].

The relationship between AlphaFold's predicted local-distance difference test (pLDDT) scores and protein flexibility is generally strong, with high pLDDT scores often indicating structurally rigid regions and low scores pointing to areas of flexibility or disorder [1], [3], [4], [5]. However, this relationship is not always straightforward. There are instances where high pLDDT scores do not necessarily equate to rigidity, as certain regions might still exhibit flexibility due to interactions with ligands or environmental conditions that are not reflected in static structural predictions [6], [7]. Similarly, low pLDDT scores may not always correspond to flexible regions, as they can also arise from the structural complexity of a region rather than inherent flexibility [1], [3].

Incorporating AlphaFold2's pLDDT scores with other methods has proven to be highly beneficial in enhancing our understanding of protein dynamics. For instance, researchers have combined pLDDT scores with molecular dynamics (MD) simulations to refine predictions of protein conformational flexibility. By using pLDDT scores to identify regions of low confidence, these regions can be targeted in MD simulations to explore potential alternative conformations or to better understand the dynamic behavior of flexible regions [2], [8]. Another approach involves integrating pLDDT scores with cryo-electron microscopy (cryo-EM) data, which allows for the validation and improvement of AlphaFold predictions by aligning the predicted structures with experimental density maps. This is particularly useful in regions with lower pLDDT scores that correspond to flexible or disordered regions in the cryo-EM maps [9]. Moreover, pLDDT scores have been used in conjunction with NMR spectroscopy data to predict protein dynamics at the residue level. By integrating these scores with NMR order parameters, researchers can estimate the flexibility of specific residues, offering insights into the dynamic features of proteins that align with experimental observations [7].

These examples demonstrate how pLDDT scores can be effectively combined with various experimental and computational techniques to gain a deeper understanding of protein dynamics, highlighting the importance of interpreting these scores alongside other data sources in structural biology research. Because of their ability to identify regions of different structural confidence, pLDDT scores are particularly useful for guiding flexibility simulations, where they can serve as distance restraints to enhance simulation accuracy [4], [7].

A decade ago, we introduced CABS-flex, a method that combines efficient coarse-grained modeling with all-atom detail for rapid simulations of protein flexibility, offering speeds three to four orders of magnitude faster than all-atom molecular dynamics (MD) simulations [10]. Since its development, CABS-flex has established itself as an efficient and cost-effective tool for simulating protein backbone flexibility across diverse biological contexts. Initially introduced as a web server (CABS-flex 1.0) [11] and later enhanced with improved features and accessibility in CABS-flex 2.0 [12], it is also available as a standalone application [13]. CABS-flex has been successfully applied to numerous structure-flexibility-function studies, including the analysis of globular proteins, mechanisms of viral adaptation, allostery, the prediction of aggregation-prone and S-nitrosylation sites, as well as structure prediction of linear and cyclic peptides [14]. For a recent review on CABS-flex applications, see [15].

In this work, we propose and test different variants of simulation constraint schemes based on pLDDT scores using the CABS-flex method. Utilizing the newly developed ATLAS database [16], which contains all-atom MD simulations of approximately 1400 proteins, along with comprehensive data such as pLDDT and RMSF, we benchmark CABS-flex performance. The diversity in protein lengths, structural domains, and secondary structure content within ATLAS makes it a valuable dataset for comparison. We compare CABS-flex simulation results, utilizing pLDDT scores, to both MD data from ATLAS and the default constraint scheme developed ten years ago, evaluating the average performance across the dataset, while also analyzing individual, particularly interesting cases.

## 2 Methods

### 2.1 Dataset

The dataset used in this study consists of 1389 PDB entries from the ATLAS database [16], which includes a diverse range of protein lengths, structural domains, and secondary structures. These PDB structures serve as direct input for CABS-flex simulations, providing experimentally validated data for modeling protein flexibility. This foundation allows the integration of pLDDT scores from AlphaFold with structural information to generate tailored restraint schemes. The MD simulations were carried out using CHARMM36m force field, with each protein simulated three times using different seeds. The initial ATLAS dataset spanned proteins from short peptides to large protein chains, but we excluded the longest case (PDB ID: 6sup_A, 2128 amino acids) due to excessively long simulation time compared to other proteins. This resulted in a final dataset spanning protein lengths from 38 to 1357 amino acids.

For optimization purposes, we extracted a training set of 400 PDB codes randomly selected from the complete dataset. The training set was used to fine-tune simulation parameters. The complete dataset was used in the final test to verify the generality of the optimized method across a wider variety of proteins.

### 2.2 Simulation engine

In this work, we utilized the CABS-flex standalone application [13], which now operates in Python 3. The CABS-flex application integrates protein dynamics simulations using the CABS coarse-grained protein model [17] with the reconstruction of selected models to all-atom representation [18] and the subsequent analysis of the modeling results. While the core simulation engine remains the same as in the previously published version, the new Python 3 wrapper enhances code management and usability. Although the updated Python 3 version of CABS-flex is still in beta and will be publicly released soon, we have made additional code available to facilitate the generation of new and improved restraint schemes, enabling users to replicate the methods described here (see Data Availability section). Given that CABS-flex utilizes Monte Carlo dynamics, the random seed serves as an essential parameter in the simulation process. To ensure complete reproducibility of the results, a fixed seed value (7503) was employed consistently across all simulations.

### 2.3 Restraints in CABS-flex

**Fig. 1:**
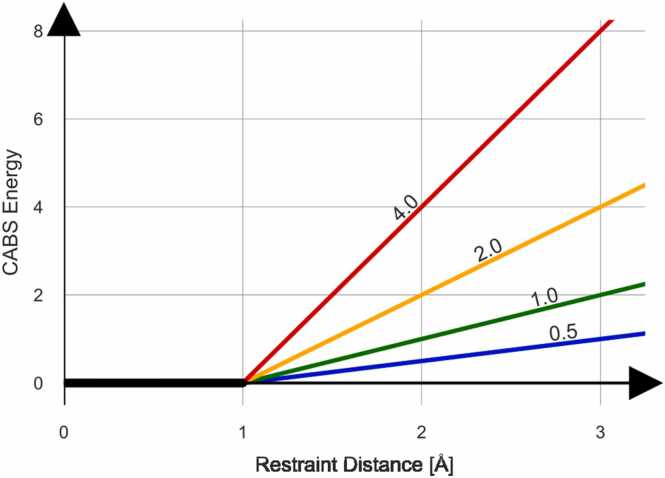
CABS energy scaling with example restraint strengths. The figure shows example restraint strengths tested in this study, color-coded according to the level of flexibility they allow: from more rigid (red), through intermediate (yellow, green), to flexible (blue). The restraint scheme adds no energy when the distance between two residues is within 1 Å of the input restraint distance (based on the input PDB). Beyond this range, the internal CABS energy increases based on Restraint Strength, with different values for distances that are too short (Restraint Strength Min) or too long (Restraint Strength Max).

CABS-flex relies on energy-based restraints that act as penalties when the distance between two residues deviates from the ideal distance defined by the input structure [10], [19]. Restraints modify the internal energy landscape, making it less likely that moves violating the restraints are accepted during Monte Carlo simulations. Two key components control the strength of restraints: min_force, which applies when the distance between residues is smaller than ideal, and max_force, which applies when the distance is larger than ideal. Fig. 1 illustrates how energy increases as the distance between restrained residues diverges from the ideal. The energy added does not fall below zero, meaning restraints only discourage non-ideal moves without biasing the model towards specific conformations. The color coding in Fig. 1 indicates varying restraint strengths, ranging from more rigid (red) to flexible (blue).

### 2.4 Root mean square fluctuations (RMSF)

To evaluate the accuracy of flexibility simulations, we used RMSF, which measures the fluctuation of each residue from its average position. For every protein in the ATLAS dataset, we compared RMSF profiles from CABS-flex simulations with those from molecular dynamics (MD) simulations. Since the ATLAS dataset includes three MD simulations for each protein, we reported the highest RMSF correlation between CABS-flex simulations and any of the three MD runs for each entry. We do not report p-values for the RMSF correlations, as they are several orders of magnitude below the 0.05 threshold, rendering significance concerns unnecessary. For evaluation of significance we used paired t-tests with p < 0.05, as implemented in the SciPy package [20]. It is important to note that, while the RMSF correlation may be high, the absolute differences between the corresponding RMSF values can still be substantial, as correlation captures local trends of increase and decrease rather than absolute magnitudes.

### 2.5 Restraint mode development

We initially evaluated three standard restraint modes in CABS-flex [12], which are widely used in both the current web server and standalone application:

• **SS2**: Restraints applied when both residues adopt regular secondary structure (alpha-helix or beta-sheet).
• **SS1**: Restraints applied when at least one residue adopts a regular secondary structure.
• **All**: Restraints applied to all residues, regardless of secondary structure.
  The SS1 and SS2 modes rely on secondary structure assignment from the DSSP algorithm, ensuring that restraints correspond to residues' structural context.
  We also developed five new restraint modes based on AlphaFold2′s predicted local-distance difference test (pLDDT) scores, which reflect confidence in the predicted structure:
• **Min Mode**:
  Applies the minimum pLDDT score from a residue pair, divided by 100, as the restraint strength. No restraints are generated if the score is below 0.5.
• **Max Mode**:
  Uses the maximum pLDDT score of the pair, following the same procedure.
• **Mean Mode**:
  Averages the pLDDT scores of the residue pair and uses this as the restraint strength.
• **pLDDT1**:
  Generates restraints if at least one of the residues in a pair has a pLDDT score above 50.
• **pLDDT2**:

Generates restraints only if both residues have pLDDT scores greater than 50.

### 2.6 Category mode

**Fig. 2:**
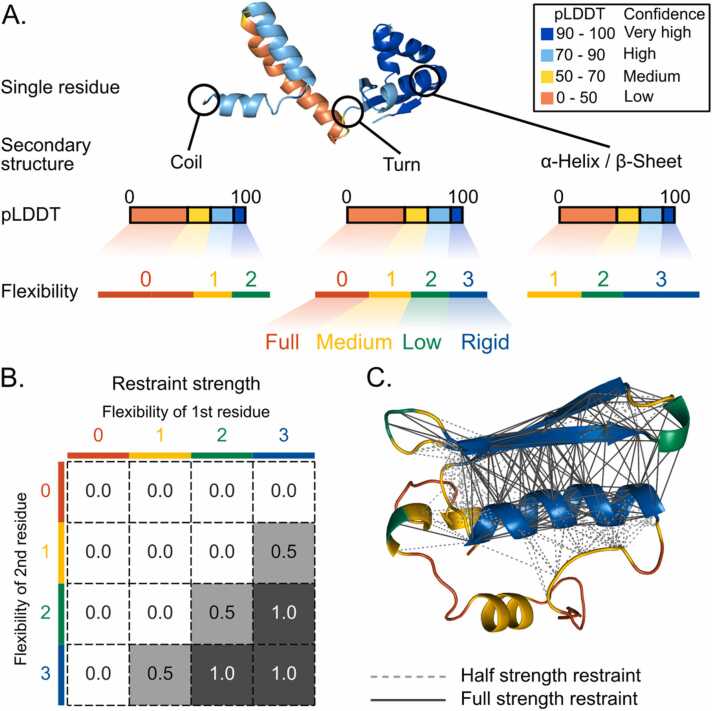
Overview of the new restraints scheme based on pLDDT scores and secondary structure. **A.** Residues are assigned to one of four flexibility categories based on a combination of their secondary structure and pLDDT scores: full flexibility (category 0), medium flexibility (category 1), low flexibility (category 2), and rigid (category 3). Residues are first classified by secondary structure into three groups: coil (most flexible), turn (moderately flexible), and alpha-helix/beta-sheet (most rigid). The pLDDT score then refines this classification, with higher confidence scores corresponding to lower flexibility for each secondary structure element. **B.** A matrix shows how restraint strength is determined based on the flexibility categories of two interacting residues. Depending on the combination of flexibility categories for the residue pair, the restraint strength can be 0.0 (no restraint), 0.5 (half strength), or 1.0 (full strength). **C.** Visualization of an example protein, highlighting how restraints are applied based on the flexibility categories assigned to different regions of the protein.

Based on initial testing, we introduced the Category mode, which combines pLDDT scores with secondary structure data. This mode assigns residues to one of four flexibility categories: fully flexible, moderately flexible, slightly flexible, and rigid. Fig. 2**A** shows how these categories are determined based on pLDDT scores and secondary structure. Fig. 2**B** shows how restraint strength is calculated based on the flexibility categories of interacting residues. Finally, Fig. 2**C** demonstrates an example of how restraints are applied to different regions of a protein.

### 2.7 Optimization of restraint parameters

**Fig. 3:**
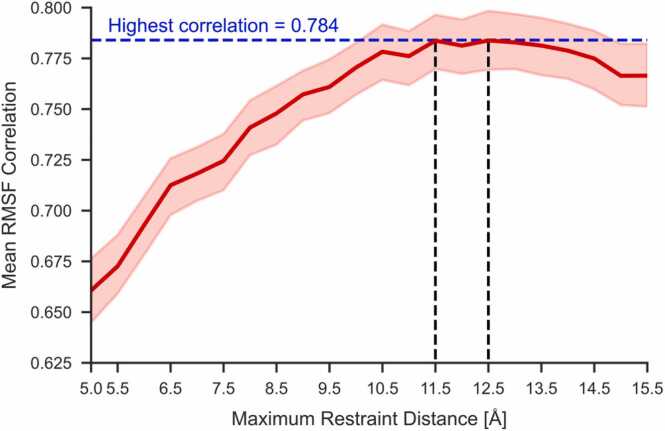
RMSF correlation between MD and CABS simulations vs Maximum Restraint distance averaged for the training set. The red borders indicate 95 % confidence intervals.

In addition to developing new modes, we optimized several key parameters to improve simulation accuracy. We tested a range of maximum restraint distances (5.0 Å to 15.5 Å, with increments of 0.5 Å) and found that a maximum distance of 11.5 Å provided the best balance between accuracy and computational efficiency (Fig. 3). We also tested different combinations of min_force and max_force, concluding that the optimal values were a min_force of 3.5 and a max_force of 0.5. This configuration provided the highest correlation with MD data, allowing for realistic flexibility simulations without excessive rigidity.

## 3 Results and discussion

The performance of the new pLDDT-based restraint modes was assessed by comparing RMSF profiles from CABS-flex simulations to those from MD simulations, using a similar approach to that of the original publication introducing the CABS-flex method [10]. In that publication, the SS2 mode (see Section 2.5) demonstrated the best performance across all tested proteins and has remained the default mode since then.

As shown in Table S1, all pLDDT-based modes outperformed the baseline SS2 mode (correlation of 0.660) in the training set. Among these, the Category mode achieved the highest average correlation with MD data, at 0.741, demonstrating that incorporating both pLDDT scores and secondary structure information leads to more accurate flexibility predictions. While the SS1 mode (with correlation of 0.712) showed improvement over SS2, the gains were relatively modest compared to the pLDDT-based modes. The Min, Max, and Mean modes produced very similar correlations of 0.731, 0.734, and 0.733, respectively, indicating that they perform better than the simpler secondary structure-based modes. The last of the standard methods - All (with a correlation of 0.734), showed comparable performance to these straightforward pLDDT modes. The pLDDT1 and pLDDT2 modes offered further small improvements, with correlations of 0.736 and 0.735. However, the Category mode consistently delivered the best performance across all tested proteins, providing the most significant improvement over the baseline SS2 mode. Its ability to classify residues using both structural confidence (pLDDT scores) and secondary structure allowed it to apply more precise and targeted restraints, thereby enhancing the model's predictive accuracy.

To further optimize performance, we investigated the impact of changing the maximum distance for applying restraints. Fig. 3 shows that increasing the maximum distance from 5.0 Å to 11.5 Å improved the correlation between CABS-flex and MD simulations. Beyond this point, performance plateaued, indicating that further increases in distance did not contribute additional benefits. Therefore, 11.5 Å was chosen as the optimal maximum distance, as it provided the best balance between accuracy and computational efficiency. Notably, optimizing this parameter resulted in the largest increase in correlation (from 0.741 to 0.784) compared to other tested parameters, highlighting its critical role in improving the overall accuracy of the simulations.

We also evaluated the effect of different sequence gaps, which define how far apart residues must be along the sequence to qualify for restraint. Although we tested all possible values from 1 to 14, Fig. S1 indicates that there was no significant improvement beyond the default gap of 3. As a result, we retained the default setting for the final simulations.

The next phase of optimization involved testing various combinations of min_force and max_force parameters. As shown in Table S4, increasing the strength of restraints, particularly max_force, reduced performance. The optimal configuration was determined to be a min_force of 3.5 and a max_force of 0.5, which allowed the model to accurately simulate flexibility while maintaining structural stability.

We also investigated whether removing a fraction of the generated restraints could maintain simulation accuracy while reducing computational demands. Fig. S2 demonstrates the effect of gradually removing restraints. The results showed that removing up to 5 % of restraints had minimal impact on performance, but further removal led to a significant decline in accuracy. This suggests that most of the restraints generated by the Category mode are necessary for maintaining realistic protein dynamics.

**Fig. 4:**
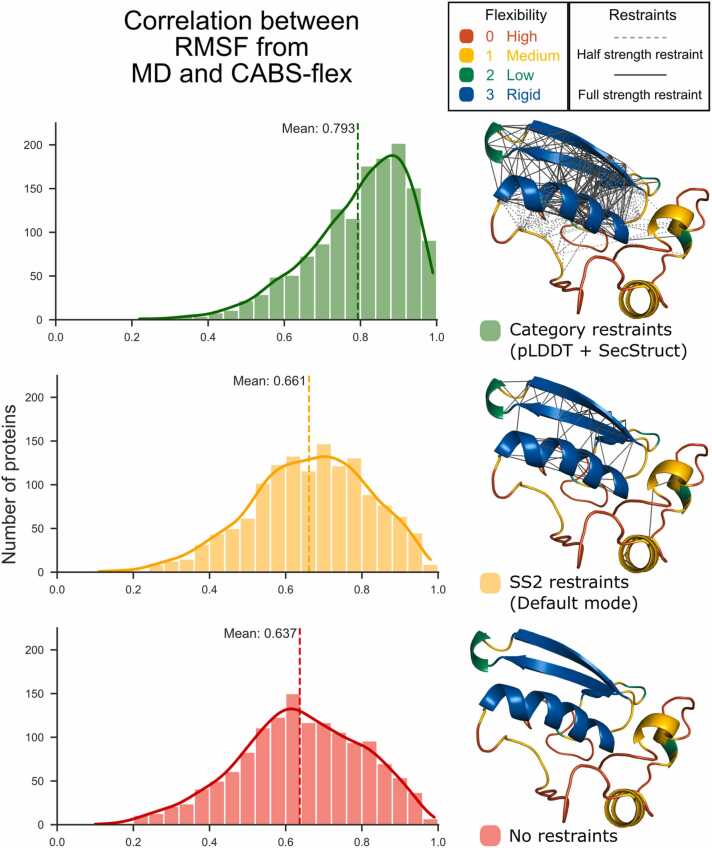
Histograms of MD and CABS-flex RMSF correlation with examples of how restraints are applied. New and optimized restraints significantly outperform the previous default and no restraints.

Finally, we compared the optimized Category mode with SS2, the previous default mode, and an approach without any restraints. As summarized in Table S6, the Category mode significantly outperformed both the SS2 mode and the no-restraint method, achieving a correlation of 0.793 across the whole dataset. Fig. 4 provides a visual comparison of RMSF correlations between these methods, along with examples of how restraints are applied to a representative protein. The histogram clearly shows the superior performance of the Category mode, which consistently achieved the highest correlation with MD simulations across a wide range of proteins. It is important to note that this correlation is observed alongside a significantly higher average RMSF in CABS-flex simulations (0.71 Å) compared to MD (0.17 Å), based on averages across the entire ATLAS dataset. This highlights the more efficient sampling provided by CABS-flex. This difference is particularly relevant given the specific dataset studied here—globular single-domain proteins—where most regions are relatively static, with only a few flexible segments. This broader sampling allows CABS-flex to complement MD by exploring conformational motions that may be undersampled in MD simulations.

These findings highlight the effectiveness of the Category mode in integrating pLDDT scores with secondary structure information, providing a more refined and accurate model for protein flexibility simulations. By optimizing parameters such as maximum distance and restraint strengths, we improved the correlation between CABS-flex and MD simulations, enhancing its accuracy in modeling the flexibility of globular proteins.

**Fig. 5:**
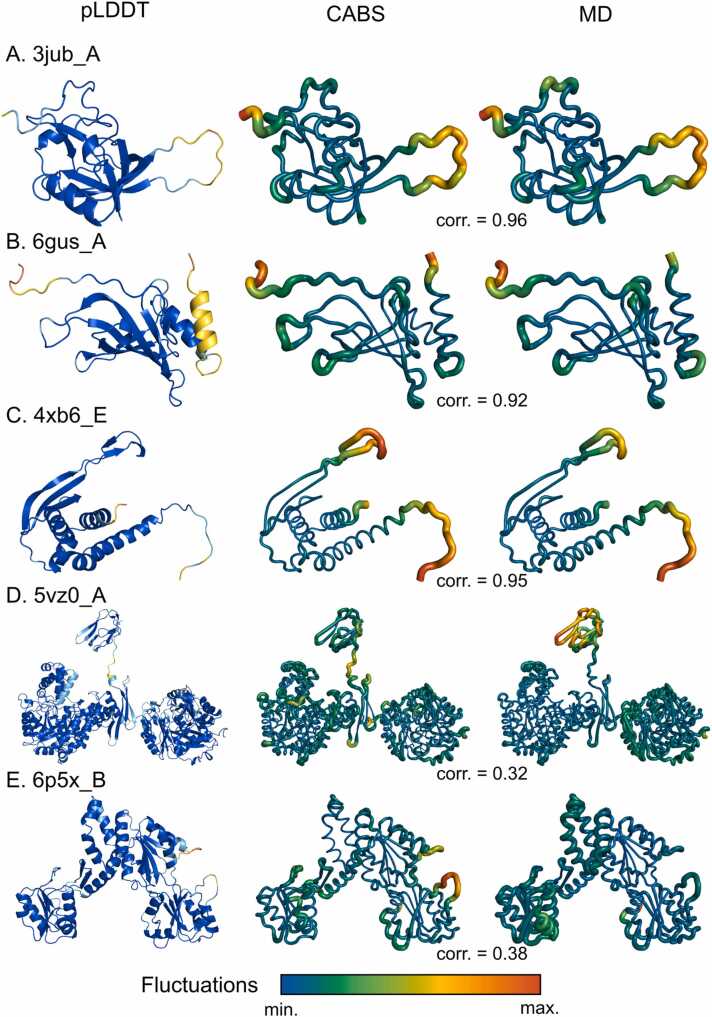
Comparison of flexibility profiles for CABS-flex and MD, visualized on experimental PDB structures. We showcase three proteins with very high correlation between CABS-flex and MD: **A.** 3jub_A; **B.** 6gus_A; **C.** 4xb6_E; and two proteins with very low correlation between CABS-flex and MD: **D.** 5vz0_A; **E.** 6p5x_E. For each protein we provide three structures (from left to right): colored by pLDDT, flexibility profile from CABS-flex simulation, and flexibility profile from molecular dynamics simulation.

To explore the performance of the Category mode in more detail, Fig. 5 presents several cases from our analysis. It illustrates experimental PDB structures colored by pLDDT scores and compares their flexibility as predicted by CABS-flex and MD, providing a direct evaluation of alignment between these methods. For most proteins in the ATLAS database, the termini are typically predicted with low confidence, as indicated by pLDDT scores. Loops are another region frequently predicted with lower pLDDT scores, as exemplified by the 3jub protein in Fig. 5**A**. Flexibility profiles from both CABS-flex and MD simulations show that the low pLDDT loop is much more dynamic than the rest of the protein. This observation aligns with electron density data from the PDB model (3jub), where the loop was unmodeled and suggested to be disordered [21]. This example illustrates that integrating pLDDT scores may help in identifying flexible regions.

However, low pLDDT scores do not always correspond to high flexibility, as shown in Fig. 5**B**. Aside from the termini, the lowest pLDDT score in this example is found in a helix. Flexibility profiles from CABS-flex and MD simulations show that this low pLDDT helix is less flexible than some loops predicted with higher confidence. This can be explained by the functional role of the helix as a linker in a protein fusion (PDB models 6gus and 6gut [22]), suggesting that it remains rigid in isolation. This highlights the importance of secondary structure information in the Category mode, as flexibility cannot be inferred from pLDDT scores alone.

In another case, seen in Fig. 5**C**, the flexibility profiles from both CABS-flex and MD simulations agree that a loop region at the top of the protein is flexible. This loop is critical for interacting with other subunits in the PDB model (4xb6) [23]. Without these subunits and stabilizing bonds, the fragment remains flexible. This example illustrates how integrating pLDDT with secondary structure helps distinguish flexible regions that might otherwise be difficult to predict accurately.

There are cases where CABS-flex diverges from MD simulations, as shown in Fig. 5**D**. This protein has three distinct domains, and except for a short linker, it is predicted with high pLDDT scores and regular secondary structure. CABS-flex predicts most of the structure to be static, which agrees with MD simulations for two of the domains. However, MD shows that the third domain is highly flexible, acting as a linker between monomers in the tetrameric complex (PDB model 5vz0) [24]. In this case, CABS-flex may overestimate rigidity, possibly due to overly strong restraints, suggesting that adjusting restraint strength could improve flexibility predictions in such cases. Conversely, in Fig. 5**E**, CABS-flex predicts more flexibility in a fragment where MD simulations suggest rigidity. Here, two adjacent loops with low pLDDT scores were unmodeled in the PDB structure (6p5x), which might explain the low scores and assumed flexibility. However, MD shows these loops remain static. This demonstrates that while CABS-flex tends to predict flexibility in regions with low pLDDT scores, this assumption does not always align with MD, particularly for loops. These discrepancies indicate that although CABS-flex generally performs well, some refinement is still needed. Overall, as shown in the histogram in Fig. 4, these misaligned cases represent mostly outliers. For the majority of proteins in the ATLAS database, CABS-flex and MD simulations align well, as demonstrated in Fig. 5**A-C**.

While our analysis incorporates experimentally validated PDB structures as input and benchmarks flexibility against MD-derived data, it does not explicitly address the relationship between pLDDT scores and experimentally observed motions captured in PDB or other experimental data. Future studies could explore this aspect to provide a more comprehensive understanding of the connection between AlphaFold-derived confidence scores and experimental observations.

## 4 Conclusions

The integration of pLDDT scores into CABS-flex through the Category mode significantly improves the alignment of protein flexibility simulations with MD results. By combining secondary structure and pLDDT information, and optimizing restraint parameters, this protocol has proven effective in better capturing protein flexibility. Our analysis shows that pLDDT scores often identify flexible regions such as loops and termini, but secondary structure is critical where pLDDT alone may not capture flexibility, such as in rigid helices or linkers. For researchers, considering pLDDT scores can be helpful in identifying flexible regions, but low pLDDT does not always indicate high flexibility, particularly in structured regions. In CABS-flex, incorporating secondary structure alongside pLDDT helps improve prediction accuracy. Additionally, adjusting restraint strength in rigid regions may further align CABS-flex results with MD simulations when discrepancies occur. Overall, the Category mode provides a reliable protocol for generating restraints, leading to more consistent flexibility predictions compared to previous methods.

Building on these findings, CABS-flex demonstrates its versatility as a simulation engine with its own force field, capable of integrating various data sources as distance restraints to guide sampling. This study establishes how pLDDT scores from AlphaFold can be incorporated into CABS-flex, offering a framework for flexibility modeling that can be extended to other structural data, such as cryo-EM NMR, or SAXS. While the current work focuses on globular single-domain proteins and comparisons with MD, which has its own sampling limitations, future adaptations could explore flexibility in more diverse protein systems, such as proteins with alternative conformational states, or incorporate experimental restraints to enhance modeling accuracy. Additionally, direct comparisons of pLDDT with experimentally observed motions remain an area for future investigation, which could further deepen our understanding of flexibility predictions.

## CRediT authorship contribution statement

**Sebastian Kmiecik:** Writing – review & editing, Writing – original draft, Supervision, Investigation, Funding acquisition, Conceptualization. **Karol Wróblewski:** Writing – review & editing, Writing – original draft, Visualization, Validation, Software, Methodology, Investigation, Formal analysis, Conceptualization.

## Declaration of Generative AI and AI-assisted technologies in the writing process

During the preparation of this work the authors used ChatGPT in order to improve readability and language. After using this tool, the authors reviewed and edited the content as needed and take full responsibility for the content of the publication.

## Declaration of Competing Interest

The authors declare no conflict of interest.

## Data Availability

Description of all the runs, result file for every run, as well as, all PDB code used in training and the whole dataset can be found on 10.5281/zenodo.13984926. The code for creating the new version of restraints can be found: https://github.com/kwroblewski7/cabsflex_restraints.
