# Supplementary material for: Integrating AlphaFold pLDDT Scores into CABS-flex for enhanced protein flexibility simulations
Source: Comput Struct Biotechnol J. 2024 Nov 30;23:4350–6. doi: 10.1016/j.csbj.2024.11.047 (PMC11653142; doi:10.1016/j.csbj.2024.11.047)
Supplement: Supplementary file 1 — Supplementary material [file mmc1.docx]

**Supplementary Information**

| Mode | Sequence Gap | Distance Min | Distance Max | Strength Min | Strength Max | MD correlation | Better than baseline^a^ |
| --- | --- | --- | --- | --- | --- | --- | --- |
| SS2 | 3 | 3.8 | 8.0 | 1.0 | 1.0 | 0.660 | Baseline |
| SS1 | 3 | 3.8 | 8.0 | 1.0 | 1.0 | 0.712 | True |
| All | 3 | 3.8 | 8.0 | 1.0 | 1.0 | 0.734 | True |
| Min | 3 | 3.8 | 8.0 | 1.0 | 1.0 | 0.731 | True |
| Max | 3 | 3.8 | 8.0 | 1.0 | 1.0 | 0.734 | True |
| Mean | 3 | 3.8 | 8.0 | 1.0 | 1.0 | 0.733 | True |
| pLDDT2 | 3 | 3.8 | 8.0 | 1.0 | 1.0 | 0.735 | True |
| pLDDT1 | 3 | 3.8 | 8.0 | 1.0 | 1.0 | 0.736 | True |
| Category | 3 | 3.8 | 8.0 | 1.0 | 1.0 | 0.741 | True |

Table S1. Comparison between different modes on mean MD correlation. ^a^p < 0.05

| Mode | Sequence Gap | Distance Min | Distance Max | Strength Min | Strength Max | MD correlation | Better than baseline^a^ |
| --- | --- | --- | --- | --- | --- | --- | --- |
| Category | 3 | 3.8 | 5.0 | 1.0 | 1.0 | 0.661 | False |
| Category | 3 | 3.8 | 5.5 | 1.0 | 1.0 | 0.673 | False |
| Category | 3 | 3.8 | 6.0 | 1.0 | 1.0 | 0.693 | False |
| Category | 3 | 3.8 | 6.5 | 1.0 | 1.0 | 0.713 | False |
| Category | 3 | 3.8 | 7.0 | 1.0 | 1.0 | 0.718 | False |
| Category | 3 | 3.8 | 7.5 | 1.0 | 1.0 | 0.725 | False |
| Category | 3 | 3.8 | 8.0 | 1.0 | 1.0 | 0.741 | Baseline |
| Category | 3 | 3.8 | 8.5 | 1.0 | 1.0 | 0.748 | False |
| Category | 3 | 3.8 | 9.0 | 1.0 | 1.0 | 0.757 | True |
| Category | 3 | 3.8 | 9.5 | 1.0 | 1.0 | 0.761 | True |
| Category | 3 | 3.8 | 10.0 | 1.0 | 1.0 | 0.771 | True |
| Category | 3 | 3.8 | 10.5 | 1.0 | 1.0 | 0.778 | True |
| Category | 3 | 3.8 | 11.0 | 1.0 | 1.0 | 0.776 | True |
| Category | 3 | 3.8 | 11.5 | 1.0 | 1.0 | 0.784 | True |
| Category | 3 | 3.8 | 12.0 | 1.0 | 1.0 | 0.781 | True |
| Category | 3 | 3.8 | 12.5 | 1.0 | 1.0 | 0.784 | True |
| Category | 3 | 3.8 | 13.0 | 1.0 | 1.0 | 0.783 | True |
| Category | 3 | 3.8 | 13.5 | 1.0 | 1.0 | 0.781 | True |
| Category | 3 | 3.8 | 14.0 | 1.0 | 1.0 | 0.779 | True |
| Category | 3 | 3.8 | 14.5 | 1.0 | 1.0 | 0.775 | True |
| Category | 3 | 3.8 | 15.0 | 1.0 | 1.0 | 0.766 | True |
| Category | 3 | 3.8 | 15.5 | 1.0 | 1.0 | 0.766 | True |

Table S2. Comparison between different distance maximums on mean MD correlation. ^a^p < 0.05

| Mode | Sequence Gap | Distance Min | Distance Max | Strength Min | Strength Max | MD correlation | Better than baseline^a^ |
| --- | --- | --- | --- | --- | --- | --- | --- |
| Category | 1 | 3.8 | 11.5 | 1.0 | 1.0 | 0.778 | False |
| Category | 2 | 3.8 | 11.5 | 1.0 | 1.0 | 0.778 | False |
| Category | 3 | 3.8 | 11.5 | 1.0 | 1.0 | 0.784 | Baseline |
| Category | 4 | 3.8 | 11.5 | 1.0 | 1.0 | 0.778 | False |
| Category | 5 | 3.8 | 11.5 | 1.0 | 1.0 | 0.774 | False |
| Category | 6 | 3.8 | 11.5 | 1.0 | 1.0 | 0.770 | False |
| Category | 7 | 3.8 | 11.5 | 1.0 | 1.0 | 0.760 | False |
| Category | 8 | 3.8 | 11.5 | 1.0 | 1.0 | 0.762 | False |
| Category | 9 | 3.8 | 11.5 | 1.0 | 1.0 | 0.760 | False |
| Category | 10 | 3.8 | 11.5 | 1.0 | 1.0 | 0.762 | False |
| Category | 11 | 3.8 | 11.5 | 1.0 | 1.0 | 0.760 | False |
| Category | 12 | 3.8 | 11.5 | 1.0 | 1.0 | 0.757 | False |
| Category | 13 | 3.8 | 11.5 | 1.0 | 1.0 | 0.758 | False |
| Category | 14 | 3.8 | 11.5 | 1.0 | 1.0 | 0.747 | False |

Table S3. Comparison between different sequence gaps on mean MD correlation. ^a^p < 0.05


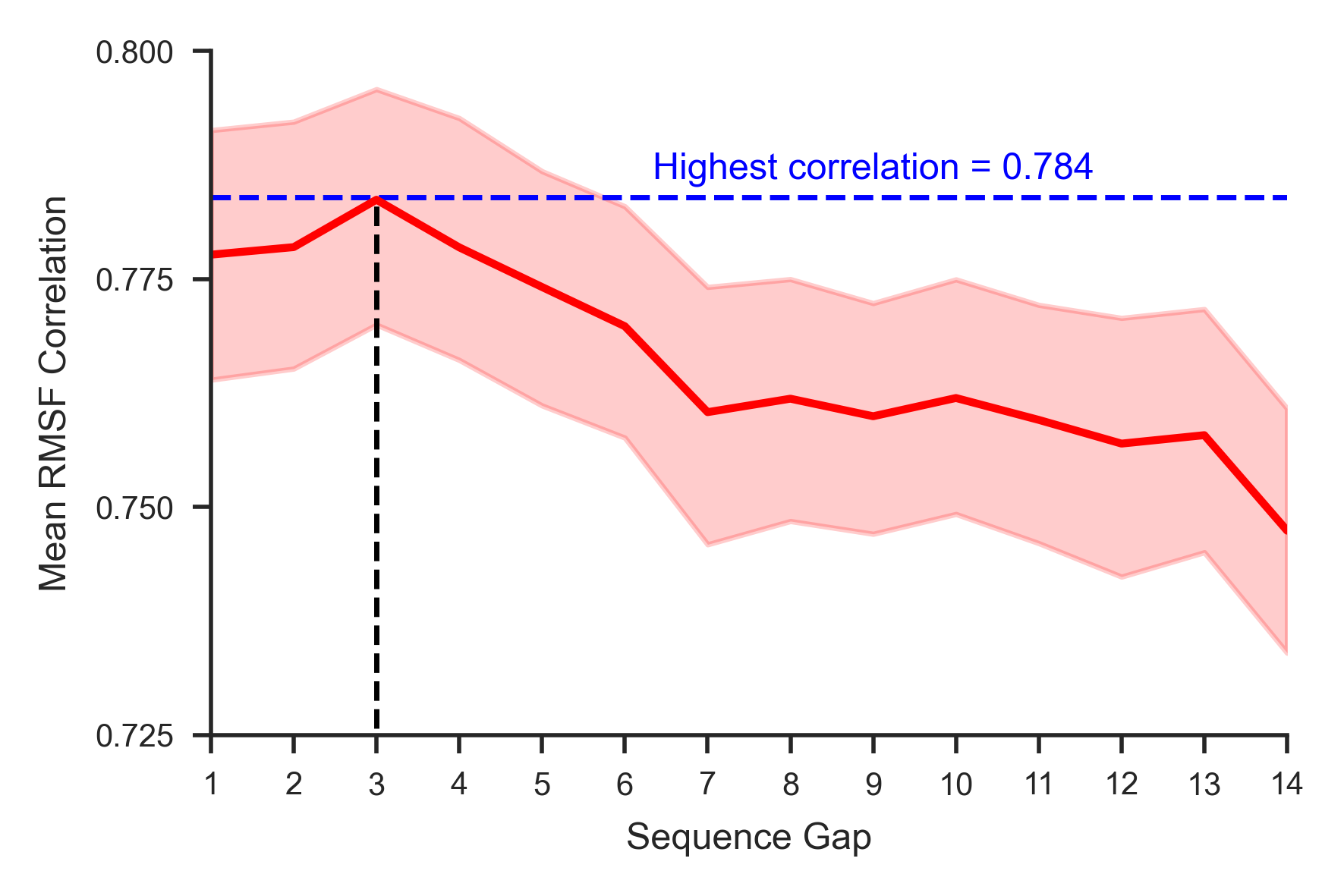


**Figure S1**. RMSF correlation between MD and CABS simulations vs Sequence Gap averaged for the training set. The red borders indicate 95% confidence intervals.

| Mode | Sequence Gap | Distance Min | Distance Max | Strength Min | Strength Max | MD correlation | Better than baseline^a^ |
| --- | --- | --- | --- | --- | --- | --- | --- |
| Category | 3 | 3.8 | 11.5 | 1.00 | 0.50 | 0.783 | False |
| Category | 3 | 3.8 | 11.5 | 1.00 | 0.75 | 0.780 | False |
| Category | 3 | 3.8 | 11.5 | 1.00 | 1.00 | 0.784 | Baseline |
| Category | 3 | 3.8 | 11.5 | 1.00 | 1.25 | 0.785 | False |
| Category | 3 | 3.8 | 11.5 | 1.00 | 1.50 | 0.780 | False |
| Category | 3 | 3.8 | 11.5 | 1.00 | 1.75 | 0.778 | False |
| Category | 3 | 3.8 | 11.5 | 1.00 | 2.00 | 0.781 | False |
| Category | 3 | 3.8 | 11.5 | 1.00 | 4.00 | 0.772 | False |
| Category | 3 | 3.8 | 11.5 | 1.00 | 8.00 | 0.770 | False |
| Category | 3 | 3.8 | 11.5 | 1.50 | 0.50 | 0.783 | False |
| Category | 3 | 3.8 | 11.5 | 1.50 | 0.75 | 0.782 | False |
| Category | 3 | 3.8 | 11.5 | 1.50 | 1.00 | 0.788 | False |
| Category | 3 | 3.8 | 11.5 | 1.50 | 1.25 | 0.782 | False |
| Category | 3 | 3.8 | 11.5 | 1.50 | 1.50 | 0.776 | False |
| Category | 3 | 3.8 | 11.5 | 1.50 | 1.75 | 0.780 | False |
| Category | 3 | 3.8 | 11.5 | 1.50 | 2.00 | 0.776 | False |
| Category | 3 | 3.8 | 11.5 | 2.00 | 0.50 | 0.791 | True |
| Category | 3 | 3.8 | 11.5 | 2.00 | 0.75 | 0.787 | False |
| Category | 3 | 3.8 | 11.5 | 2.00 | 1.00 | 0.789 | False |
| Category | 3 | 3.8 | 11.5 | 2.00 | 1.25 | 0.782 | False |
| Category | 3 | 3.8 | 11.5 | 2.00 | 1.50 | 0.777 | False |
| Category | 3 | 3.8 | 11.5 | 2.00 | 1.75 | 0.778 | False |
| Category | 3 | 3.8 | 11.5 | 2.00 | 2.00 | 0.775 | False |
| Category | 3 | 3.8 | 11.5 | 2.00 | 4.00 | 0.770 | False |
| Category | 3 | 3.8 | 11.5 | 2.00 | 8.00 | 0.766 | False |
| Category | 3 | 3.8 | 11.5 | 2.50 | 0.50 | 0.791 | True |
| Category | 3 | 3.8 | 11.5 | 2.50 | 0.75 | 0.779 | False |
| Category | 3 | 3.8 | 11.5 | 2.50 | 1.00 | 0.781 | False |
| Category | 3 | 3.8 | 11.5 | 2.50 | 1.25 | 0.783 | False |
| Category | 3 | 3.8 | 11.5 | 2.50 | 1.50 | 0.779 | False |
| Category | 3 | 3.8 | 11.5 | 2.50 | 1.75 | 0.773 | False |
| Category | 3 | 3.8 | 11.5 | 2.50 | 2.00 | 0.775 | False |
| Category | 3 | 3.8 | 11.5 | 3.00 | 0.50 | 0.788 | False |
| Category | 3 | 3.8 | 11.5 | 3.00 | 0.75 | 0.780 | False |
| Category | 3 | 3.8 | 11.5 | 3.00 | 1.00 | 0.782 | False |
| Category | 3 | 3.8 | 11.5 | 3.00 | 1.25 | 0.777 | False |
| Category | 3 | 3.8 | 11.5 | 3.00 | 1.50 | 0.778 | False |
| Category | 3 | 3.8 | 11.5 | 3.00 | 1.75 | 0.779 | False |
| Category | 3 | 3.8 | 11.5 | 3.00 | 2.00 | 0.770 | False |
| Category | 3 | 3.8 | 11.5 | 3.50 | 0.50 | 0.792 | True |
| Category | 3 | 3.8 | 11.5 | 3.50 | 0.75 | 0.784 | False |
| Category | 3 | 3.8 | 11.5 | 3.50 | 1.00 | 0.786 | False |
| Category | 3 | 3.8 | 11.5 | 3.50 | 1.25 | 0.776 | False |
| Category | 3 | 3.8 | 11.5 | 3.50 | 1.50 | 0.781 | False |
| Category | 3 | 3.8 | 11.5 | 3.50 | 1.75 | 0.776 | False |
| Category | 3 | 3.8 | 11.5 | 3.50 | 2.00 | 0.777 | False |
| Category | 3 | 3.8 | 11.5 | 4.00 | 0.50 | 0.788 | False |
| Category | 3 | 3.8 | 11.5 | 4.00 | 0.75 | 0.783 | False |
| Category | 3 | 3.8 | 11.5 | 4.00 | 1.00 | 0.785 | False |
| Category | 3 | 3.8 | 11.5 | 4.00 | 1.25 | 0.779 | False |
| Category | 3 | 3.8 | 11.5 | 4.00 | 1.50 | 0.776 | False |
| Category | 3 | 3.8 | 11.5 | 4.00 | 1.75 | 0.775 | False |
| Category | 3 | 3.8 | 11.5 | 4.00 | 2.00 | 0.770 | False |
| Category | 3 | 3.8 | 11.5 | 4.00 | 4.00 | 0.763 | False |
| Category | 3 | 3.8 | 11.5 | 4.00 | 8.00 | 0.755 | False |
| Category | 3 | 3.8 | 11.5 | 8.00 | 1.00 | 0.774 | False |
| Category | 3 | 3.8 | 11.5 | 8.00 | 2.00 | 0.765 | False |
| Category | 3 | 3.8 | 11.5 | 8.00 | 4.00 | 0.757 | False |
| Category | 3 | 3.8 | 11.5 | 8.00 | 8.00 | 0.743 | False |

Table S4. Comparison between different restraint strengths on mean MD correlation. ^a^p < 0.05

| Mode | Sequence Gap | Distance Min | Distance Max | Strength Min | Strength Max | Fraction of restraints removed | MD correlation | Better than baseline^a^ |
| --- | --- | --- | --- | --- | --- | --- | --- | --- |
| Category | 3 | 3.8 | 11.5 | 3.5 | 0.5 | 0.00 | 0.792 | Baseline |
| Category | 3 | 3.8 | 11.5 | 3.5 | 0.5 | 0.01 | 0.787 | False |
| Category | 3 | 3.8 | 11.5 | 3.5 | 0.5 | 0.02 | 0.783 | False |
| Category | 3 | 3.8 | 11.5 | 3.5 | 0.5 | 0.03 | 0.786 | False |
| Category | 3 | 3.8 | 11.5 | 3.5 | 0.5 | 0.04 | 0.790 | False |
| Category | 3 | 3.8 | 11.5 | 3.5 | 0.5 | 0.05 | 0.793 | False |
| Category | 3 | 3.8 | 11.5 | 3.5 | 0.5 | 0.10 | 0.784 | False |
| Category | 3 | 3.8 | 11.5 | 3.5 | 0.5 | 0.15 | 0.785 | False |
| Category | 3 | 3.8 | 11.5 | 3.5 | 0.5 | 0.20 | 0.782 | False |
| Category | 3 | 3.8 | 11.5 | 3.5 | 0.5 | 0.25 | 0.783 | False |
| Category | 3 | 3.8 | 11.5 | 3.5 | 0.5 | 0.30 | 0.785 | False |
| Category | 3 | 3.8 | 11.5 | 3.5 | 0.5 | 0.35 | 0.780 | False |
| Category | 3 | 3.8 | 11.5 | 3.5 | 0.5 | 0.40 | 0.775 | False |
| Category | 3 | 3.8 | 11.5 | 3.5 | 0.5 | 0.45 | 0.772 | False |
| Category | 3 | 3.8 | 11.5 | 3.5 | 0.5 | 0.50 | 0.773 | False |
| Category | 3 | 3.8 | 11.5 | 3.5 | 0.5 | 0.60 | 0.757 | False |
| Category | 3 | 3.8 | 11.5 | 3.5 | 0.5 | 0.70 | 0.743 | False |
| Category | 3 | 3.8 | 11.5 | 3.5 | 0.5 | 0.80 | 0.720 | False |
| Category | 3 | 3.8 | 11.5 | 3.5 | 0.5 | 0.90 | 0.681 | False |
| No restraints | N/A | N/A | N/A | N/A | N/A | 1.00 | 0.635 | False |

Table S5. Comparison between different fraction of restraints removed on mean MD correlation. ^a^p < 0.05


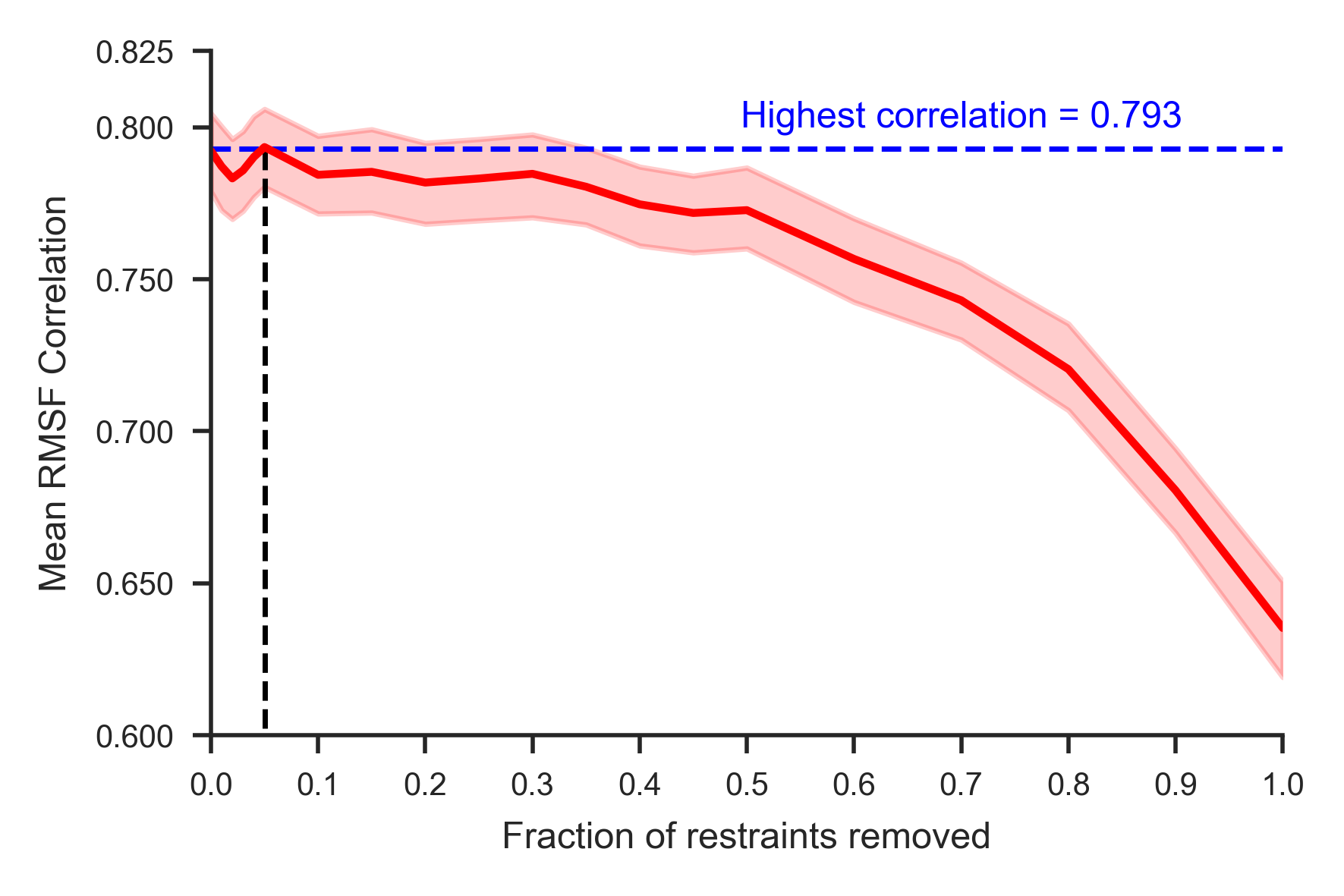


**Figure S2**. RMSF correlation between MD and CABS simulations vs Fraction of restraints removed averaged for the training set. The red borders indicate 95% confidence intervals.

| Name | Mode | Sequence Gap | Distance Min | Distance Max | Strength Min | Strength Max | MD correlation |
| --- | --- | --- | --- | --- | --- | --- | --- |
| pLDDT + SecStruct | Category | 3 | 3.8 | 11.5 | 3.5 | 0.5 | 0.793 |
| Previous default | SS2 | 3 | 3.8 | 8.0 | 1.0 | 1.0 | 0.660 |
| No restraints | No restraints | N/A | N/A | N/A | N/A | N/A | 0.635 |

Table S6. Overall comparison between new and optimized restraints, previous default restraints, and no restraints on mean MD correlation.
